# Twelve tips for addressing ethical concerns in the implementation of artificial intelligence in medical education

**DOI:** 10.1080/10872981.2024.2330250

**Published:** 2024-04-03

**Authors:** Russell Franco D’Souza, Mary Mathew, Vedprakash Mishra, Krishna Mohan Surapaneni

**Affiliations:** a Department of Education, UNESCO Chair in Bioethics, Melbourne, Australia; b Department of Organisational Psychological Medicine, International Institute of Organisational Psychological Medicine, Melbourne, Australia; c Department of Pathology, Kasturba Medical College, Manipal, Manipal Academy of Higher Education (MAHE), Manipal, India; d School of Hogher Education and Research, Datta Meghe Institute of Higher Education and Research (Deemed to be University), Nagpur, India; e Department of Biochemistry, Panimalar Medical College Hospital & Research Institute, Chennai, India; f Department of Medical Education, Panimalar Medical College Hospital & Research Institute, Chennai, India

**Keywords:** Artificial intelligence, ethics, ethical concerns, medical education, medical educators

## Abstract

Artificial Intelligence (AI) holds immense potential for revolutionizing medical education and healthcare. Despite its proven benefits, the full integration of AI faces hurdles, with ethical concerns standing out as a key obstacle. Thus, educators should be equipped to address the ethical issues that arise and ensure the seamless integration and sustainability of AI-based interventions. This article presents twelve essential tips for addressing the major ethical concerns in the use of AI in medical education. These include emphasizing transparency, addressing bias, validating content, prioritizing data protection, obtaining informed consent, fostering collaboration, training educators, empowering students, regularly monitoring, establishing accountability, adhering to standard guidelines, and forming an ethics committee to address the issues that arise in the implementation of AI. By adhering to these tips, medical educators and other stakeholders can foster a responsible and ethical integration of AI in medical education, ensuring its long-term success and positive impact.

## Practice points

- In the ever-evolving landscape of medical education, the integration of Artificial Intelligence (AI) stands out as a revolutionary innovation with the potential to reshape learning methodologies and advance healthcare practices.
- However, this transformative journey is impeded by ethical concerns that demand careful attention.
- This reflects a delicate balance that educators must strike between embracing innovation and ensuring responsible implementation.
- The twelve provided tips serve as a practical guide, highlighting the complexities involved in incorporating AI ethically.
- By following these guidelines, educators contribute to shaping a healthcare workforce that is not only technologically proficient but also ethically grounded.

## Introduction

With the emergence of Artificial Intelligence (AI), various domains including medical education are anticipated to undergo massive transformations in several key areas such as personalized learning experiences, enhanced diagnostic training, offering students a hands-on experience without risk; augmented decision-making skills, fostering deeper understanding and clinical reasoning; and streamlined administrative tasks, allowing educators to focus more on teaching and less on paperwork [1]. As the discussions over the integration of AI into medical education delve deeper, it becomes imperative for educators to fully understand the implications of AI and the potential concerns over its responsible use [2]. AI undoubtedly can revolutionize medical education by facilitating and improving personalized learning, instant access to a wide range of information, technology-enhanced teaching, adaptive assessments, immediate contextual feedback, collaborative learning through virtual platforms and support faculty in creating advanced, data-driven curricula to stay aligned with the dynamic landscape of education [3]. However, along with these transformative benefits, AI also raises significant ethical concerns including privacy issues related to the handling and security of sensitive student and patient data, biases inherent in AI algorithms that could lead to unequal educational opportunities or misinterpretations in clinical training, the lack of transparency in AI decision-making processes making it difficult for users to understand how conclusions are drawn, and the potential devaluation of human judgment as AI becomes more integrated into educational frameworks [4]. Even though AI has numerous advantages, medical schools worldwide are yet to explore AI to its full potential with robust implementation. The main reasons for this include the lack of AI expertise in faculty and the lack of standard guidelines for the responsible use of AI [5]. Hence, for seamless integration of AI in medical education and improved quality of education, as envisioned in the United Nations Sustainable Development Goals [6], it becomes increasingly important that educators understand the ethics of AI in medical education to guarantee high-quality learning, guide students in responsible AI use, address biases, advocate for transparent and ethical AI practices and fostering fairness in education. In light of this, we recommend the following twelve tips for addressing the ethical concerns in the use of AI in medical education that educators can consider to promote the effective and responsible use of AI.

### Tip 1: ensuring transparency in the development and deployment of AI systems in medical education

Transparency in context of AI refers to openness and clarity in the development, functioning, and deployment of artificial intelligence systems [7]. In medical education, transparency is crucial to building trust among educators, students, and other stakeholders to understand how the labyrinthine network of AI works and arrive at decisions [8]. Transparent AI systems enable the comprehension of the logic behind recommendations and assist in the continuous improvement of educational content [9]. To begin with, choose AI systems with explainable algorithms. This allows educators to understand and communicate to students how the AI system reaches specific conclusions or recommendations. Also, it is important to maintain documentation on the development and deployment of AI systems. This documentation should include the data used, the training process, and the decision-making logic of the AI algorithms. However, it’s essential to acknowledge that complete transparency in AI systems is not achievable in all contexts. There are inherent limitations due to the complexity of AI algorithms, especially concerning general intelligence in AI and the potential for proprietary or sensitive information. This acknowledgement does not undermine the pursuit of transparency but rather sets a realistic expectation for the levels of understanding and insight that can be provided.

Imagine a medical school implementing an AI-powered adaptive learning platform designed to personalize educational content based on individual student needs. In this scenario, transparency would involve openly communicating how the AI system operates. The institution should provide the expected information on the algorithms used, the data sources feeding into the system, and how decisions are made. Clear communication about the goals and expected outcomes of the AI implementation helps students and educators understand the purpose of the technology and dispels any concerns about hidden biases or opaque decision-making processes. By being transparent, the institution establishes a foundation for trust, promoting a positive environment for the integration of AI in medical education.

### Tip 2: addressing biases in AI algorithms to ensure fair representation of educational content

Biases in AI algorithms can lead to unequal representation and impact the quality of medical education. This bias in AI algorithms can manifest in various ways such as selection or information bias, potentially leading to unfair and inaccurate outcomes [10]. AI’s reliance on knowledge derived from available literature and experimental results underscores the importance of a strong foundation in the data and information used to train or develop AI systems [11]. Hence, the quality and diversity of the input data play a significant role in shaping the system’s knowledge base as well as the output derived [12]. Considering this, a new term ‘Algorithmic fairness’ has been introduced. It seeks to minimize biases by scrutinizing data during preprocessing, optimizing throughout algorithm development, and assessing the output during postprocessing [13]. Hence, it is important to ensure training data is diverse and representative to avoid biases in algorithmic outcomes. Also, it is recommended to conduct bias audits regularly to identify and rectify any biases present in the AI system. However, just like humans, machines also have implicit bias and eliminating all bias in AI systems may not be fully achievable, but it is imperative to strive towards minimizing biases to ensure fairness and equity in AI applications.

Consider an AI-driven recommendation system for medical study materials. To ensure fair representation, the algorithm should include diverse data sets and must undergo regular audits for biases related to gender, ethnicity, and socioeconomic factors. For instance, if the algorithm consistently recommends certain topics more to one demographic, it may indicate bias. Institutions should actively engage in bias detection and correction processes to guarantee that the AI system provides equal opportunities for learning to all students, regardless of their background or characteristics. This commitment to fairness strengthens the educational experience and aligns with the principles of diversity and inclusion.

### Tip 3: validating the output generated by AI educational tools to ensure the accuracy and credibility of information

Validating the content generated by AI-based educational tools is essential to uphold the accuracy and credibility of information provided to students and educators. This process involves subjecting the AI-generated content to rigorous evaluation by domain experts and educators [14,15]. Additionally, encouraging feedback from end-users, such as students and educators, establishes a continuous improvement loop. This ensures that the AI-generated content aligns with current evidence-based medical knowledge and educational standards [16]. Validation is not a one-time process but a dynamic and ongoing effort to maintain the quality and reliability of educational content delivered through AI tools [17]. This commitment to accuracy contributes to the effectiveness of AI in enhancing the learning experience in medical education [18].

Suppose an AI system is responsible for creating medical case studies for students. In this context, rigorous validation processes would involve subjecting the generated content to review by medical experts. By engaging medical professionals to review and possibly revise the material, potential inaccuracies, outdated information, or misleading content can be identified and corrected. This validation ensures that the information presented aligns with current medical knowledge, guidelines, and ethical standards. By implementing such validation procedures, institutions can confidently incorporate AI-generated content into their curriculum, providing students with accurate and reliable educational materials. This not only enhances the learning experience but also instills a sense of aplomb in the use of AI in medical education. Although it is a time-consuming process, this process also prevents passive usage or dependance on AI systems for content creation.

### Tip 4: prioritize privacy with robust measures to protect student and patient data used in AI-driven educational tools

Safeguarding the privacy of student and patient data is paramount when integrating AI-driven educational tools [19]. This tip underscores the need for robust measures to ensure the confidentiality and security of sensitive information. Implementation of advanced data encryption techniques plays a crucial role in protecting individual identities and maintaining the integrity of personal and health-related data [20,21]. Anonymizing data whenever feasible adds an extra layer of privacy, allowing educational insights without compromising individuals’ identities [22]. Thus, adhering to strict security protocols, such as secure infrastructure and access controls, establishes a protective framework. By prioritizing privacy-preserving AI tools, educational institutions can instil trust among students, educators, and patients, fostering a secure and ethical environment for AI-driven medical education [23]. Even though complete privacy in AI models cannot be guaranteed because of changing risks and complex data issues, the aim should be set to keep information as safe as possible.

However, anonymization is not the only way to protect user data always. In instances where student data will be used for educational research or when the overall performance of the model is considered, data anonymization is important. However, imagine an AI system that analyzes student performance data to provide personalized feedback. In this scenario, data anonymization may not be possible. Users should be willing to sacrifice a certain level of privacy to enjoy the benefits of personalization. By clearly communicating the benefits of data used for personalization, along with the measures taken to protect sensitive information, ensure confidentiality and reduce the risk of unauthorized access, institutions can build the trust of their users. However, balancing privacy with personalization requires a dynamic approach, adjusting policies and practices as technologies and societal expectations evolve.

By demonstrating a commitment to privacy, the institution safeguards both student data and compliance with regulations such as the Health Insurance Portability and Accountability Act (HIPAA). This is designed to protect the privacy of individuals’ health information while allowing the flow of health information needed to ensure high-quality health care and to protect the public’s health and well-being [24]. By adhering to HIPAA, institutions can reinforce the ethical use of AI in medical education.

### Tip 5: obtaining informed consent from students and stakeholders regarding the use of AI technologies in educational processes

Consent is a key component in respecting individuals’ autonomy and fostering a collaborative and ethical educational ecosystem where all stakeholders are informed participants in the adoption of AI technologies [25]. Obtaining written informed consent is a foundational step in the ethical integration of AI technologies into educational as well as clinical processes [26]. This tip emphasizes the importance of transparent communication, acceptance and collaboration with students and stakeholders. Obtaining explicit consent ensures that individuals are aware of and agree to the use of AI tools in their educational experiences. This process should involve limpid explanations of how AI technologies will be utilized, the purpose of their integration, and any potential impact on the learning environment [27].

Suppose an educational institution decides to implement an AI-powered learning analytics platform that tracks students’ online learning behaviors, such as time spent on modules, engagement with course materials, assessment, remedial recommendation and patterns of interaction with the learning management system. The institution would need to clearly articulate that the AI analytics tool aims to enhance the overall learning experience by providing personalized insights into individual study habits, identifying areas of strength and weakness, and offering tailored recommendations for improvement. Also, the institution should provide students with the option to either opt-in or opt-out of the AI-enhanced learning analytics. This empowers students to make informed decisions based on their comfort level with data collection and AI-driven personalization.

### Tip 6: fostering collaboration between AI experts, educators, and students to bring diverse perspectives and expertise

Encouraging collaboration among AI experts, educators, and students is integral for a holistic and inclusive approach to the integration of AI in education [28,29]. By facilitating open communication and teamwork, this tip ensures that technical expertise from AI professionals is combined with pedagogical insights from educators and the firsthand experiences and expectations of students [30]. Involving students in these discussions provides valuable input into the development process, helping to tailor AI applications to meet their needs and preferences [31]. This collaborative model not only enriches the educational experience but also empowers students to actively engage with AI technologies, fostering a sense of ownership and responsibility [32].

Imagine a collaborative effort between a medical school, AI researchers, and students to develop an AI-driven virtual escape room for emergency medical scenarios. AI experts contribute their knowledge in natural language processing to create realistic visuals and game dynamics. Educators provide insights into emergency clinical scenarios and treatment strategies, ensuring the tool aligns with curriculum goals. Students can actively participate in discussions about game features, factors that motivate them, level of difficulty and feedback sessions offering perspectives on the AI-based escape room authenticity, gaming experience and educational value. This collaboration results in a powerful educational tool that enhances students’ skills and decision-making in emergency clinical settings.

### Tip 7: conducting faculty development programs on AI ethics to equip them with the knowledge and skills necessary to guide students

Ensuring that educators are well-versed in AI ethics is crucial for responsible and informed integration of AI technologies into education [33]. This underscores the importance of offering specialized training to educators, equipping them with the knowledge and AI competencies needed to utilize the full potential and navigate ethical considerations associated with AI [34]. Training sessions should cover topics such as bias mitigation, privacy protection, and transparency in AI algorithms [35]. This proactive approach not only prepares educators for the challenges posed by AI but also cultivates a culture of ethical awareness and responsibility among both educators and students [36].

For instance, if an educational institution plans to train their medical students in using Clinical Decision Support Systems (CDSS), educators who will be involved in training the students must be aware of the functioning and ethical concerns in using CDSS such as patient data privacy, transparency, bias, accuracy, consent etc. If educators are not aware, it not only leads to improper and irresponsible use of CDSS for teaching purposes but also paves the way for students to misuse CDSS in future without having proper guidelines on its ethical use.

### Tip 8: providing student education on AI and its implications in medical education

Empowering students with comprehensive knowledge about the role of AI in medical education is essential for fostering a prescient and informed AI-based learning environment [37]. This tip emphasizes the importance of integrating AI literacy into the curriculum to ensure that students are well-versed in the applications, benefits, and potential challenges associated with AI technologies in the medical field. This also proactively prepares students to embrace the opportunities presented by AI in their clinical practice while fostering a sense of responsibility in its ethical and effective use [38,39].

Consider a medical college introducing AI in radiology. Here, students should undergo training on AI applications that expose them to an AI-driven diagnostic imaging tool, emphasizing hands-on experience on its functioning, scope of performance, costs, and other pros and cons. Through workshops, students can gain proficiency in interpreting medical images with AI assistance. The curriculum should also stress ethical considerations, such as biases in training data and transparency in algorithms. Collaboration with own or other hospitals employing AI in radiology can provide real-world insights on its effective implementation and use. This approach ensures that future health professionals are not only technically adept but are also ethically conscious users of AI technologies in medical imaging [40].

### Tip 9: ensuring ongoing maintenance of AI algorithms for performance, accuracy, and reliability

This tip emphasizes the technical and operational side of AI usage in medical education. The focus here is on the continual evaluation of AI systems to ensure they function as intended, remain accurate over time, and are reliable for educational purposes. Continuous monitoring and assessment of AI algorithms are essential for maintaining their effectiveness, accuracy, and reliability in medical education [41]. The rapidly evolving nature of medicine highlights a significant challenge when relying on AI systems: if these systems are based on algorithms constructed several years ago, they may become outdated and less effective. Hence, periodic assessments should be conducted to check for accuracy in generating educational content, ensuring that the information remains up-to-date and aligns with current medical knowledge [42]. Additionally, monitoring the algorithm’s performance helps identify any potential biases or unintended consequences that may arise during its use [43]. Ethical considerations, such as patient and student privacy, should also be a focal point of these assessments [44].

For instance, establishing a feedback loop involving educators, AI developers, and stakeholders through surveys and personal in-depth interviews regarding AI performance, user satisfaction, feasibility, accuracy, relevance and educational impact ensures that adjustments can be made promptly based on real-world usage and evolving ethical standards. By embracing this approach to monitoring and assessment, institutions can enhance the reliability and ethical use of AI algorithms, contributing to a continually improving and responsible educational environment.

### Tip 10: establishing clear lines of accountability for the development, deployment, and outcomes of AI systems in medical education

Defining clear lines of accountability is paramount when integrating AI systems into medical education [45]. Assigning specific roles to AI developers, educators, administrators, and other stakeholders including students ensures that everyone understands their responsibilities and is accountable for the ethical and effective use of AI in education [46]. This accountability extends to addressing potential biases, ensuring data privacy, and monitoring the impact of AI on the learning experience [47]. Establishing such a transparent framework for accountability not only mitigates risks but also fosters a culture of responsibility and collaboration among all involved parties [48].

Consider an instance where a medical university implements an AI-based tutoring system to assist medical students in learning complex topics. Clear accountability should be established, designating the IT department for data security, the curriculum committee for educational alignment, and a dedicated AI oversight team for monitoring outcomes. This clear delineation of responsibilities ensures that the tutoring system meets educational goals, maintains data security, and is continuously improved based on feedback.

### Tip 11: enhancing the regulatory awareness about the standards governing the use of AI in healthcare and medical education

In the upcoming phases of integrating AI into healthcare and medical education, it is imperative for institutions to stay vigilant, informed, and compliant with pertinent regulations and standards [49]. The institutions should stay informed about healthcare data protection laws, such as HIPAA, and ensure the AI system complies with these regulations [50]. Regular updates and compliance checks should be conducted to adapt to changes in laws, thereby maintaining legal and ethical integrity in the use of AI for healthcare and medical education [51].

Looking ahead, as the landscape of AI in healthcare continues to evolve, institutions are expected to proactively track and adhere to emerging regulations governing the ethical and secure use of AI technologies [52]. This forward-looking approach ensures that the integration of AI into medical education aligns with evolving legal frameworks, thereby safeguarding patient data, ensuring privacy, and upholding the ethical standards essential to responsible AI deployment in healthcare settings [53]. By staying informed and complying with these evolving regulations, institutions can position themselves to contribute positively to the advancement of medical education through the ethical and lawful integration of AI technologies [54,55].

### Tip 12: forming an ethics committee or engage existing institutional review boards for the ethical oversight of AI in medical education

Creating dedicated ethics committees or involving existing institutional review boards is pivotal in proactively addressing any ethical issues that may arise in the implementation of AI in medical education [56]. Though not martinet, the ethics committees play a crucial role in stressing the importance of adherence to regulation and scrutinizing potential ethical challenges, including privacy concerns, fairness, and transparency [57]. In addition to regulatory considerations, the ethics committee role also involves considering the legal concerns that extend into the realm of liability, compliance with broader laws beyond specific regulations, and the implications of AI decisions, particularly when things go wrong [58]. This includes understanding the potential legal consequences of AI failures or malfunctions, and who bears the responsibility in such situations. Engaging institutional review boards leverages their expertise in ethical review processes, particularly when handling sensitive data or novel applications of AI [59]. Regular collaboration with these ethical oversight bodies ensures that any emerging ethical concerns are identified and addressed promptly [60]. This approach not only safeguards against potential ethical pitfalls but also contributes to the establishment of ethical best practices, promoting responsible and accountable use of AI in medical education [61]. This tip addresses the ethical governance and oversight of AI usage. While, as discussed previously, Tip 9 is more about the ‘mechanical’ aspects of AI – its functioning, accuracy, and reliability after the implementation, as a part of ongoing evaluation, while this tip focuses on the ‘moral’ and ‘legal’ dimension, dealing with the ethical implications and standards governing AI use in educational settings before and after the implementation, ensuring AI’s use aligns with moral and ethical standards, and institutional policies.

For instance, consider a scenario where a medical school is planning to implement an AI-driven simulation for surgical training. In preparation, the institution must form an ethics committee composed of medical professionals, educators, and AI experts. This committee is tasked with reviewing the simulation’s content for accuracy, fairness, and appropriateness. They also assess potential psychological impacts on students participating in the simulations, ensuring a holistic ethical evaluation. The institution, through its ethics committee or IRB, should also establish protocols for reporting any biases, inaccuracies, or unintended consequences observed during AI tool utilization. This reporting mechanism will serve as a feedback loop, allowing continuous improvement and addressing ethical issues promptly.

## Conclusion

The integration of Artificial Intelligence in medical education holds immense promise, offering transformative benefits such as personalized learning and advanced curricular development. However, the realization of this potential is hindered by ethical concerns that demand proactive attention from educators and stakeholders. The twelve tips outlined here serve as a roadmap for educators, institutions, officials and policy makers, empowering them to harness the potential of AI while upholding ethical standards. As medical educators embrace these principles, they not only elevate the quality of teaching and learning but also shape a future healthcare workforce that is not just technologically proficient but ethically grounded. By integrating these ethical guidelines into the integration of AI in medical education, educators play a pivotal role in ensuring the responsible and sustainable evolution of AI, ultimately benefiting both current and future generations of healthcare professionals.
